# Impact of the COVID-19 pandemic on cancer assessment in primary care: a qualitative study of GP views

**DOI:** 10.3399/BJGPO.2021.0056

**Published:** 2021-06-02

**Authors:** Stephanie Archer, Natalia Calanzani, Stephanie Honey, Margaret Johnson, Richard Neal, Suzanne E Scott, Fiona M Walter

**Affiliations:** 1 Research Associate, Department of Public Health and Primary Care, University of Cambridge, Cambridge, UK; 2 Research Associate, Leeds Institute of Health Sciences, University of Leeds, Leeds, UK; 3 Patient/Public Partner, The Primary Care Unit, Department of Public Health and Primary Care, University of Cambridge, Cambridge, UK; 4 Professor of Primary Care Oncology, Leeds Institute of Health Sciences, University of Leeds, Leeds, UK; 5 Senior Lecturer in Health Psychology, Faculty of Dentistry, Oral and Craniofacial Sciences, King’s College London, London, UK; 6 Reader in Primary Care Cancer Research, The Primary Care Unit, Department of Public Health and Primary Care, University of Cambridge, Cambridge, UK

**Keywords:** general practice, neoplasms, early diagnosis, coronavirus

## Abstract

**Background:**

Early diagnosis is key to improve cancer outcomes, and most cancers are diagnosed in primary care after initial symptomatic presentation. Emerging evidence suggests an increase in avoidable cancer deaths owing to the COVID-19 pandemic.

**Aim:**

To understand GPs’ views on the impact of the COVID-19 pandemic on the clinical assessment of possible cancer.

**Design & setting:**

A qualitative semi-structured interview study with GPs from the East of England.

**Method:**

GPs were purposively sampled based on age, sex, and years of experience. Interviews were conducted via Zoom or Microsoft Teams in August and September 2020. Transcribed recordings were analysed inductively using thematic analysis. The Model of Pathways to Treatment guided the analysis.

**Results:**

Three themes were identified across 23 interviews on GP views on the impact of: (1) changes in patient help-seeking behaviour on symptoms at presentation; (2) remote consultations on managing patients with possible cancer symptoms; and (3) the COVID-19 pandemic on triaging and referring patients with possible cancer. There were positive changes to practice, but concerns were raised about the adequacy of remote consultations for assessing symptoms. Some GPs reported delayed cancer diagnoses, and uncertainty about how backlog in referrals would be managed.

**Conclusion:**

This study provides new evidence on the impact of the COVID-19 pandemic on assessing symptomatic patients. Recommendations are made to inform safe and effective primary care clinical practice. Urgent action is needed to mitigate the impact of the COVID-19 pandemic, and ensure appropriate symptomatic assessment now and in the future.

## How this fits in

In order to understand whether and how the COVID-19 pandemic affected clinical assessment of possible cancer in primary care, semi-structured interviews were conducted with GPs. GPs described an impact of patient help-seeking on symptoms at presentation, sometimes leading to presenting with more advanced symptoms. The benefits and challenges of remote consultations were explored. Many felt they are not always appropriate to assess cancer symptoms and signs. The pandemic had also impacted testing, triaging, and referring patients with possible cancer. Recommendations are made to ensure safe and effective symptomatic assessment in primary care now and in the future.

## Introduction

Cancer is the leading cause of death in many countries, and its burden and prognosis are highly dependent on disease stage at diagnosis.^1^ In countries such as England, with a gatekeeper healthcare system, up to 90% of patients with cancer are diagnosed after initial symptomatic presentation in primary care. Recent policy, research, and clinical strategies have focused on promoting early presentation of patients to primary care and reducing diagnostic delays with timely referral by GPs to specialty care.^2–4^

It is feared that the COVID-19 pandemic has dramatically affected all aspects of the patient’s pathway to diagnosis with cancer and subsequent treatment services.^2,4–6^ In March 2020, England entered its first national lockdown. Before the emergency, most GP consultations occurred in person whereas, within a week of lockdown, there was an almost universal shift from face-to-face consultations to email, telephone, or video consultations.^7–10^ Huge reductions were initially detected in the demand for primary care services, and these did not fully recover even with the easing of lockdown.^11^ The population is now accessing primary care in a different way,^10^ and often staying away from medical services.^7^ This is likely to have implications for timely presentation of symptoms suggestive of cancer if patients postpone presentation in primary care. There are also likely to be implications for primary care detection of cancer owing to the impact of remote consultations on clinical assessment,^2^ and the process and timescales for referral to specialist services,^9,12,13^ but these have not yet been reported.

Consequences for cancer‐related burden and mortality are inevitable and likely to be long lasting.^14^ Near real-time data on cancer care and cancer deaths suggest that the COVID-19 pandemic may contribute, over a 1-year time horizon, to substantial excess mortality among people with cancer and multimorbidity.^15^ Few data have yet emerged of public and healthcare professional views on the impact of the COVID-19 pandemic on clinical assessment of possible cancer in primary care. Indeed, the rapid changes in medical practice necessitated by the pandemic may add value to care, and create opportunities going forward. Interviews with GPs were undertaken that aimed to understand their perspectives regarding the impact of the COVID-19 pandemic on their clinical assessment of possible cancer, in order to make recommendations to ensure safe and effective future clinical practice for primary care.

## Method

### Approach

Semi-structured interviews were used to explore the views of GPs and patients recruited from primary care in the East of England via the existing FIT-East study.^16^ Results from the patient interviews are reported separately.

### Participants

GPs were invited to participate via the East of England Clinical Research Network (CRN); they were sampled to achieve a spread of age, sex, and years of experience. Interviews continued until the topics discussed did not contribute new themes to the analysis.

### Data collection

Health services researchers with expertise in qualitative research (SA and SH) conducted the interviews, which took place via Zoom or Microsoft Teams at a time of the GP’s choosing, following informed consent. All interviews were completed between 7 August and 10 September 2020. The interview topic guide was developed from the earlier study exploring the use of the faecal immunochemical test (FIT) in primary care, updated to explore GPs’ experiences of caring for patients during the COVID-19 pandemic (see supplementary data file 1).

### Analysis

Verbatim transcripts were checked, anonymised, and analysed inductively using thematic analysis.^17^ In order to become familiar with the data, two researchers (SA and NC [a health services researcher]) repeatedly read all the transcripts and generated initial codes. Formal coding, using the Dedoose platform,^18^ involved working through the transcripts to identify and label relevant extracts. Coding was informed by the Model of Pathways to Treatment^19,20^ in order to place the evidence within the context of intervals in the cancer pathway (symptom appraisal, help-seeking, diagnosis, and pre-treatment), while recognising the roles of patients, the GPs, and the healthcare system. The codes were drawn together into a broad set of initial themes; consistency of coding between SA and NC was discussed regularly through team meetings, and a third researcher was consulted if necessary (SH). Themes were further refined with guidance from senior team members (FW and RN [academic GPs], and SS [health psychologist]).

## Results

Twenty-three GPs from 16 different practices were interviewed (see Table 1); 13 were male. GPs varied in age (34 to 62 years) and experience (<1 year to 35 years). Most GPs (*n* = 15) worked at practices with up to 10 000 patients and described their practice as being in an urban setting (*n* = 11).

**Table 1.** Demographic characteristics of the GP sample

Demographic	Category	*n* (%)
Sex	Male	13 (57)
	Female	10 (43)
Setting	Rural	5 (22)
	Semi-rural	5 (22)
	Urban	11 (48)
	Missing	2 (8)
Age, years	30–40	5 (22)
	41–50	6 (26)
	51–60	7 (31)
	≥61	2 (8)
	Missing	3 (13)
Years of GP experience	0–5	1 (4)
	6–10	6 (26)
	11–20	8 (35)
	≥21	7 (31)
	Missing	1 (4)
Teaching or training role	Yes	14 (61)
	No	9 (39)
Number of patients registered at practice	0–5000	0 (0)
	5001–10 000	15 (66)
	10 001–15 000	2 (8)
	15 001–20 000	5 (22)
	≥20 001	1 (4)

Almost all GPs described how they had to change their personal practice because of the COVID-19 pandemic, including their experience of working from home, an increased workload, and having to get used to remote instead of face-to-face consultations (something they believed would continue in the long term). Several GPs described their need to shield or work remotely to protect their health or the health of their loved ones, whereas others recognised that they were exposing themselves to risk.

Some GPs described pre-existing health system challenges that were highlighted by the COVID-19 pandemic, such as limited numbers of consulting rooms and shortage of clinicians. Several GPs mentioned the effect of the COVID-19 pandemic on primary care earnings such as Quality and Outcome Framework (QOF) payments. Several GPs believed that the COVID-19 pandemic would continue to influence service provision in the upcoming year. Others described a future of uncertainty in primary care.

Three main themes were identified on GP views on the impact of: (1) changes in patient help-seeking behaviour on symptoms at presentation; (2) remote consultations on managing patients with possible cancer symptoms; and (3) the COVID-19 pandemic on triaging and referring patients with possible cancer. These themes are presented, with quotations contextualised with the participant’s ID number, sex, and years of GP experience.

### Changes in patient help-seeking behaviour impacted symptoms at presentation

#### Mismatch between patient expectations and practice availability

GPs reported that demand for appointments was reduced when lockdown started. Demand then slowly increased over time or even became higher than it was before the COVID-19 pandemic. Some GPs described a mismatch between patient views and expectations of primary care provision and what was available to them. There were reported misconceptions about practices being closed or GPs being too busy with COVID-19-related activities (often attributed to inaccurate reports from the media). Several GPs emphasised that primary care services remained available, even during lockdown, and that their practice was 'always open':

'*I think if they had presented to us during COVID times with the symptoms, they would have been seen, but they didn’t know that we were open or that the services were available.* *'* (GP07, F, 6–10 years)

*'Well, that’s not true, we’ve not been closed at all and in fact, we’ve been working really hard because we’ve had to change quite a lot* […] *we’ve been open all the time and we’ve always been accessible to our patients and we’ve made it quite clear that we still want to see people particularly with possibly cancer symptoms.* *'* (GP13, F, 11–20 years)

#### Patients’ increased health vigilance yet reluctance to seek help as a result of the COVID-19 pandemic

While a few reported uncertainty over whether patients’ help-seeking behaviour had changed, some GPs discussed how the COVID-19 pandemic had resulted in patients exhibiting increased health vigilance by taking more ownership and personal responsibility for their health:

*'I think the threats* [that] *COVID presented to patients actually made them re-think, engage in some more home care and self-care to a certain degree,* […] *so, I think there’s been some adaptation or change in terms of* [help-] *seeking behaviour.* *'* (GP22, M, 0–5 years)

However, key discussions referred to patient reluctance to seek help and reported consequences for symptom presentation and GPs' symptom assessments. Several GPs stated that patients seemed reluctant to seek help owing to the risk of catching COVID-19. GPs described how patients often made a conscious decision to delay presentation. Some questioned whether patients did not present because they were told to stay at home where possible, or were excessively worried about COVID-19 owing to available public information:

*'They didn’t want to trouble us because of COVID so they waited a couple of months and then a couple of months again because it didn’t seem to be growing that much, and then you have proper cancer, basically.'* (GP11, F, 11–20 years)

Some GPs gave further accounts of patients who had presented with more advanced symptoms or even tumours:

*'So, patient presenting… with … malignancy-type symptoms: we’ve had a couple who were quite advanced because they’ve held off from presenting during COVID.'* (GP23, F, 6–10 years)

*'I see basal cell carcinomas twice as big as they would normally be, because they have been waiting for 9 months, and the waiting list is now ridiculous.'* (GP11, F, 11–20 years)

*'I did have a patient who’s had a neck lump for nearly5months and didn’t tell us at all* […]*. Yes, and this gentleman has sort of a tumour* *…* *'* (GP08, M, 6–10 years)

Some GPs were concerned about delays in detecting early cancer for timely treatment. Importantly, a few believed that the usual approach to 'watch and wait' may not always be appropriate, particularly when symptoms could be owing to cancer, as the disease may already be advanced:

*'The point with cancer care is you have to diagnose at a very early stage…my concern… is that we are missing… those early cancers* [as] *… only the investigation tells because their symptoms are so vague that you cannot pick* [them] *up otherwise* *.* *..* *'* (GP15, M, 11–20 years)

*'You just have to be really extra vigilant really with any sort of presentation at the moment, because it may be a late presentation of something. So it probably does mean we’re investigating a lot more patients, whereas maybe* [previously] *we may have had a ‘wait and watch’ approach* *.'* (GP10, M, 6–10 years)

### Impact of remote consultations on managing patients with possible cancer symptoms

#### Less able to assess subtle symptoms and signs

Most GPs discussed the pros and cons of remote versus face-to-face consultations. While most acknowledged being able to continue to provide full GP services via remote consultations, for some GPs, patients *'* *just need to be seen* *'* face to face. Several GPs expressed a personal preference for face-to-face consultations. One GP described it as *'* *good medicine* *'*, others as a chance to have a personal touch and better understand the patient. They described how face-to-face consultations allowed them to *‘* *look the patient in the eye* *’*, note more subtle signs of possible cancer, and allow patients to report additional symptoms; for example, the *‘* *conversation as patients leave the consulting room* *’*:

*'The classic* [is] *when someone* *… speaks to you* *… about their neck pain and then at the end says, oh, by the way, whilst I’m here, I’m just a bit worried about this lump in my breast* *… Those sort of things we perhaps aren’t getting so much* [of] *because we haven’t got that same interaction with them.* *'* (GP13, F, 11–20 years)

*'I think, if you have got somebody in the room with you, it’s far easier to eyeball people and say, you’re well, you’re ill.'* (GP12, F, 11–20 years)

Some GPs talked about the usefulness of ‘gut feeling’ during face-to-face contact with a patient, which is known to be an important factor when managing cancer uncertainty:

*'[I]n terms of assessing people on the phone, it’s just not quite the same as seeing somebody facetoface and, sort of, thinking “you’re ill, you’ve got something going on, I’m not sure what.” And yeah, you sort of lose another way of assessing people. Of course, it’s not perfect, but you do get the impression when somebody walks in as to…that they’ve got something going on that you’ve got to get to the bottom of.*' (GP09, M, ≥21 years)

*'You miss when somebody walks in the room you think “this is something serious” sometimes, that is what you don’t have on the telephone. So you need to have those people in or visit them to make sure you still can make those sort of judgments.'* (GP14, F, ≥21 years)

Several GPs gave examples of when a face-to-face consultation was still required, such as persistent or ongoing symptoms, and physical symptoms that raised a suspicion of cancer. This was particularly the case for bowel symptoms and patients with abdominal pain, when a physical examination was required:

*'If you think they have some form of malignancy, you still have to bring them to the surgery, make the assessment, and then do the onward referral to the secondary care.'* (GP08, M, 6–10 years)

*'Most of the people we’ve seen facetoface, even in the COVID times, have* [had] *abdominal pain* […] *Even if you say it’s not serious, most people want to put* *…* *or I personally want to put my hand on the tummy to just make sure there are no signs of an acute abdomen or something* *.* *..* *'* (GP18, M, 11–20 years)

*'I think if you’ve got even the slightest concern about a carcinogenic process, you need to see them facetoface. I don’t think you can really avoid it. So normally after a telephone consultation or a telephone call, if their symptoms are persisting you want to see them facetoface.'* (GP17, M, ≥21 years)

GPs also spoke about challenges with using video consultations, including the time it took to set them up and limited patient access to reliable internet connection or to a smartphone, and how this affected certain patient groups who may be at higher risk of cancer.

#### Less able to manage cancer uncertainty

Half the GPs described their role managing risk, believing that not being able to have face-to-face consultations resulted in taking more risks and potentially missing signs that could indicate cancer. At times, this increased risk was reported to cause anxiety among GPs:

*'Relying less on what we see, and just trusting more what we hear* […] *It’s come with its own level of anxiety as well, if you’re ever anxious of making the wrong decision, this last* *6* *months has been the time.* *'* (GP22, M, 0–5 years)

*'I think, there’s undoubtedly a greater degree of risk, be it perceived or be it real, with doing more remote consulting.'* (GP12, F, 11–20 years)

A couple of GPs highlighted the importance of safety netting to mitigate the increased risk:

*'I think the changes have forced us to carry a bit more of the weight of responsibility and uncertainty than we used to. But as a GP, that’s what you do, you’re faced with uncertainty all the time…* (GP21, M, 6–10 years)

*'We’ve all been a bit more uncomfortable with the perceived increase[d] risk, and you’re having to do more safety netting, you’re having to do more advice on when to return and all of those things, and who to seek help from. So, I think, it is more risky.'* (GP12, F, 11–20 years)

### Impact of the COVID-19 pandemic on triaging and referring patients with possible cancer

#### Re-purposing diagnostic resources, using the FIT as an exemplar

Some GPs highlighted that guidance around referrals was constantly changing. For example, in 2017, the National Institute for Health and Care Excellence (NICE) diagnostics guidance DG30 recommended that FITs should be requested routinely for all patients with non-specific gastrointestinal or vague symptoms, as a positive FIT demonstrated faecal haemoglobin and recommendation for an urgent referral and colonoscopy. During May and June 2020, many clinical commissioning groups (CCGs) began to recommend extending use of FIT to patients meeting the NICE suspected cancer guideline NG12 owing to limited access to colonoscopies. Several but not all the interviewees were aware of this new requirement for a FIT result before referring a patient via the 2-week wait (2WW) urgent pathway:

*'[C]olonoscopies and gastroscopies were stopped during COVID…* [then] *you had to do a FIT test. Even if you were trying to refer to a* *2* *-week wait you required a FIT test, and only if that was positive, then they would go ahead and do a colonoscopy.* *'* (GP05, M, ≥21 years)

Some GPs also discussed using FITs more often, sometimes resulting in shortages. Some believed that FIT had an extra use as patients did not need to be seen face to face, consistent with reducing COVID-19 risk:

*'I think it’s a valuable tool. It’s so easy to do for patients. It’s non-invasive. It’s done pretty remotely. They can send it in, and they can do it in the privacy of their own home. So it’s not even bringing patients in to expose them to our healthcare assistants.'* (GP05, M, ≥21 years)

*'Again, you know, when it’s sometimes even difficult to see patients face to face, arranging for them to do a FIT test could be very helpful.'* (GP01, M, ≥21 years)

Some GPs commented on the utility of FIT as a diagnostic test, suggesting that it has good performance data, is useful for 'debatable' cases, and could reassure both healthcare professionals and patients:

*'Our threshold for using a FIT test has dropped and, even for those grey-area patients… we’re probably more likely to do a FIT test* [now] *than we were pre-COVID, because it* *… provides very useful, hopefully reassuring, information.* *'* (GP01, M, ≥21 years)

While some GPs stated that they had started to order tests before seeing the patient, another believed that testing should not be used as a substitute for other types of assessment:

*'[W]hat I personally do is, people with abdominal symptoms who contact me, I tend to take the history over the phone. And again, it’s about trying to limit contact with them too, because of COVID, some people I will send to test first, because* […] *I know they’re going need a FIT. Then sometimes even without examining them, I will speak to them over the phone, take a thorough history and if they’re fulfilling the criteria for it and not meeting the* *2* *-week wait threshold, which excludes FIT, then I’ll send them for screening blood tests and the FIT at the same time, and then follow them up afterwards. So, some people you need to examine, but some people you don’t before you send them to test.* *'* (GP12, F, 11–20 years)

*'I think it’s a bad principle to do tests on somebody who you haven’t fully… assessed. Doing tests is part of your assessment but when I qualified we were taught 70%is in the story, 20%is in the examination and 10%in the test* […] *I’m definitely ordering fewer tests because* […] *I’m less likely to order a test if I haven’t actually seen somebody face to face. I don’t see a test as a substitute for seeing them.* *'* (GP03, M, ≥21 years)

#### Interconnectedness between NHS primary and secondary care: changes in secondary care impact primary care

GPs talked about both positive and negative ways in which changes in secondary care had impacted primary care. They reported positive impacts of the COVID-19 pandemic on diagnosing patients, including using advice from secondary care or meeting with specialists to discuss diagnostic multidisciplinary team (MDT) decisions for their patients:

*'We’ve used a lot of advice and guidance with the hospital system, and that’s actually worked really well …Previously advice and guidance was fairly useless, in that it would take a couple of months to get any response back, whereas now* [within] *48* *hours* *you get a message back.* *'* (GP09, M, ≥21 years)

However, most GPs believed that cancer diagnoses had been delayed as a result of COVID-19 and described delays in secondary care investigations, particularly for routine and outpatient services. For instance, there were reports of cancelled or postponed endoscopies and imaging tests:

*'We used to be able to book routine CT abdo pelvis* [but] *the routines are still not being done. So that has introduced delays and every so often you get a nasty shock with one of those, or your vague suspicion would be confirmed.* *'* (GP21, M, 6–10 years)

GPs also described how patients waiting for secondary care appointments continued to seek support from primary care to prevent their condition from deteriorating. One GP talked about the need to contact patients when appointments due to a suspicion of cancer were cancelled:

*'And quite a lot of them coming back to us with problems which are kind of worsening in the meantime, and we’ll be trying to firefight and trying to establish which of those people actually have deteriorated and need to be sort of pushed up the line.'* (GP13, F, 11–20 years)

*'*[we were] *given a list of patients whose scans were cancelled because you booked them as a routine, and then you have to go back and talk to them about how they’re feeling now.'* (GP07, F, 6–10 years)

Some GPs were concerned that patients could be fearful about attending hospital appointments owing to concerns about COVID-19 risk:

*'And I had …occasional situations where… somebody seemed unwell, where you might want them in the hospital, where you might want to refer them, and they said, “no, not going anywhere near that hospital, because I’ll get corona.”'* (GP20, F, 11–20 years)

One GP stated that cancer treatment was also being delayed, while a few others described the increased use of telephone or remote appointments in secondary care. There were particular concerns about how secondary care would be able to manage large waiting lists and an increasing backlog, in terms of how long this would take and whether there were enough resources to do so:

*'The dermatology department has got a 42-weekwait at the moment…they’ve always been short of clinicians anyway, but the waiting list is massive.'* (GP19, F, ≥21 years)

## Discussion

### Summary

Interviews during late summer 2020 with GPs in the East of England demonstrated their concerns that the COVID-19 pandemic has had a significant impact on clinical assessment for possible cancer in primary care. They focused on three areas of the patient pathway to cancer diagnosis, namely the impact of: changes in patient help-seeking behaviour on symptoms at presentation; remote consultations on managing patients with possible cancer symptoms; and the COVID-19 pandemic on triaging and referring patients with possible cancer. While some changes were generally viewed as positive, most GP participants raised concerns that these rapid changes in medical practice, necessitated by the pandemic, may have negatively impacted safe and effective management in primary care, and potentially led to delays in cancer diagnoses.

Building on these findings and published literature,^4,5,7–10,12,13,21^ a list of key recommendations have been developed for practice (see Box 1) in order to provide safe, effective, and high quality care for patients with a suspicion of cancer or other serious condition during the current and future pandemics.

Box 1 **Recommendations for practice**

- Reassure patients that primary care is ‘open’ as an accessible and safe environment. To enable this, messaging from the NHS, the government, and the media needs to be consistent, age-appropriate, and involve multiple communication approaches (television, radio, newspapers, and so on).
- Recognise patients’ reluctance to seek help across the health and social care spectrum (for example, vulnerable populations) and actively enquire about vague or persistent symptoms.
- Value face-to-face consultations for examinations, including in care homes and on home visits, to ensure equitable high quality care.
- Have a low threshold to use blood, urine, and imaging tests in primary care and in patients’ homes to enhance assessment of risk of cancer, particularly when examination is limited or not possible. Inflammatory markers and faecal immunochemical tests (FITs) can be considered for wider use.
- Adopt a proactive approach to safety netting, including follow-up telephone calls and booked appointments.
- Prioritise excellent communication with patients and with colleagues across primary and secondary care settings.
- Maintain cancer care services in a safe secondary care environment to facilitate patient attendance and timely assessment and treatment, in order to prevent backlog and subsequent increased workload in primary care.

### Comparison with existing literature

Consistent with NHS data,^11^ the findings suggest the perceived availability of services initially impacted on how patients navigated health and illness during the COVID-19 pandemic, with fewer patients accessing primary care. While this drop in healthcare use provided an opportunity for some to self-manage and resolve minor symptoms themselves,^7^ for others it may have resulted in later presentation with symptoms suggestive of cancer.^9,12,13^ GPs in the study linked the reluctance to consult to patients’ fears of catching COVID-19.^2,4,5,9^ The COVID-19 pandemic was a catalyst for change in primary care and increased the use of remote consultations. Concerns about quality and safety of remote consultations raised by other authors were also confirmed by interview findings.^4,7–9,21–23^ While remote consulting can be useful, it may not always be safe, particularly when assessing patients with symptoms that may indicate cancer. The importance of safety netting in this context has been highlighted by others,^5,9^ alongside the need to be vigilant when patients take longer to present.^9^ The findings are also in line with expectations that the COVID-19 pandemic would affect different steps in the diagnostic pathway.^4^ Interviews indicate this is likely to be the case owing to backlog in imaging and delayed routine surgeries.^2,9,12,13^ This not only delayed timely treatment, but also increased workload in primary care as GPs supported patients with ongoing symptoms.

### Strengths and limitations

To the authors' knowledge, this is the first study to report the views of English GPs about the impact of the COVID-19 pandemic on their clinical assessment of possible cancer, collected via interviews conducted between the first and second waves of the pandemic in late summer 2020. There was good variation among interviewees in terms of age, sex, and years of GP experience. The sample was limited in terms of geographical location, which may reduce the generalisability of the findings across other regions of England, the other nations in the UK, and other countries with similar ‘gatekeeper’ healthcare systems such as Denmark and the Netherlands. A strong conceptual framework was used — the Model of Pathways to Treatment — to underpin not only the framing of the interview schedule but also the organisation of the data, in order to take a systematic approach to understand the events and processes that were affected by the COVID-19 pandemic.

While GPs gave their views on patient experiences and a range of influences on their timely help-seeking, it is important to understand the views of patients themselves. Interviews were conducted with patients in the same study, and the findings are reported separately. These focus mainly on the ‘patient interval’, when patients appraised their bodily changes and symptoms, and made decisions about when to seek help from their GPs. This study benefited from patient and public involvement at every stage: MJ is a patient partner on the project. Finally, while this study focused on clinical assessment for possible cancer in primary care, many of the findings will be relevant for detecting and diagnosing other chronic conditions.^24^

### Implications for research and practice

GPs and allied healthcare professionals will need to make the most of available, and often scarce, resources to effectively meet the needs of patients with a suspicion of cancer in a way that is also safe, managing both cancer and COVID-19 risk. In a context where both remote and face-to-face consultations are the norm, the recommendations will need universal adoption in order to provide equitable and consistent high quality care. Increased use of safety netting is particularly important in this new scenario.

There is also a key role for policymakers to ensure that both primary and secondary care professionals have adequate and equitable access to safe environments for examinations, and use of biomarker and imaging tests to triage patients with possible symptoms of cancer. Good quality tests that can provide reassurance are particularly important when there is more uncertainty and GPs need to manage increased risk.

Policy highlighting the need for, and importance of, safe and effective clinical practice is particularly urgent, specifically regarding the use of remote consultations. Improving communication and the interface between primary and secondary care is also essential for early cancer diagnosis. This may require reorganisation of local, regional, and national levels and systems, and developing and evaluating different pathways to cancer diagnosis.

Studies that investigate the impact of remote consulting on quality and safety of care are required,^7^ specifically exploring the building of trust and rapport.^9^ The potential impact of remote consultations on exacerbating healthcare disparities,^7,9,22,25–27^ particularly among older and vulnerable populations, also needs to be further investigated. There will be an increasing role for studies incorporating implementation science and evaluation in order to assess existing and new practices and modes of care. Finally, more clinical studies investigating the use of non-invasive cancer diagnostic tests in primary care populations are urgently needed. Such tests may be particularly useful to address the backlog of investigations in secondary care, even if a short-term alternative.^28^

In conclusion, this study adds to the body of evidence indicating the impact of the COVID-19 pandemic on timely diagnosis of cancer from GP perspectives, and provides recommendations to reduce the future burden of late cancer diagnosis. While some changes to primary care accelerated by the COVID-19 pandemic have had a positive impact, there are several important concerns to be addressed so patients with a suspicion of cancer can be safely assessed in primary care.

## References

[1] De Angelis R, Sant M, Coleman MP (2014). Cancer survival in Europe 1999–2007 by country and age: results of EUROCARE--5—a population-based study. Lancet Oncol.

[2] Hamilton W, Walter FM, Rubin G, Neal RD (2016). Improving early diagnosis of symptomatic cancer. Nat Rev Clin Oncol.

[3] Rubin G, Berendsen A, Crawford SM (2015). The expanding role of primary care in cancer control. Lancet Oncol.

[4] Helsper CW, Campbell C, Emery J (2020). Cancer has not gone away: a primary care perspective to support a balanced approach for timely cancer diagnosis during COVID‐19. Eur J Cancer Care.

[5] van Weert H (2020). After the first wave: what effects did the COVID-19 measures have on regular care and how can general practitioners respond to this?. Eur J Gen Pract.

[6] The Lancet Oncology (2020). UK cancer care threatened by government incompetence. Lancet Oncol.

[7] Khan N, Jones D, Grice A (2020). A brave new world: the new normal for general practice after the COVID-19 pandemic. BJGP Open.

[8] Gray DP, Freeman G, Johns C, Roland M (2020). Covid 19: a fork in the road for general practice. BMJ.

[9] Jones D, Neal RD, Duffy SRG (2020). Impact of the COVID-19 pandemic on the symptomatic diagnosis of cancer: the view from primary care. Lancet Oncol.

[10] Park S, Elliott J, Berlin A (2020). Strengthening the UK primary care response to covid-19. BMJ.

[11] NHS Digital (2020). Appointments in general practice September 2020. https://digital.nhs.uk/data-and-information/publications/statistical/appointments-in-general-practice/september-2020.

[12] Maringe C, Spicer J, Morris M (2020). The impact of the COVID-19 pandemic on cancer deaths due to delays in diagnosis in England, UK: a national, population-based, modelling study. Lancet Oncol.

[13] Sud A, Torr B, Jones ME (2020). Effect of delays in the 2-week-wait cancer referral pathway during the COVID-19 pandemic on cancer survival in the UK: a modelling study. Lancet Oncol.

[14] Richards M, Anderson M, Carter P (2020). The impact of the COVID-19 pandemic on cancer care. Nat Cancer.

[15] Lai AG, Pasea L, Banerjee A (2020). Estimated impact of the COVID-19 pandemic on cancer services and excess 1-year mortality in people with cancer and multimorbidity: near real-time data on cancer care, cancer deaths and a population-based cohort study. BMJ Open.

[16] CanTest (2018). Establishing acceptability and outcomes for faecal immunochemical tests (FITs) in the English primary care symptomatic population: a mixed-methods study in the East of England. https://www.cantest.org/research-projects/east-of-england/.

[17] Braun V, Clarke V (2006). Using thematic analysis in psychology. Qual Res Psychol.

[18] Dedoose Version 8.0.35 (2018). Web application for managing, analyzing, and presenting qualitative and mixed method research data.

[19] Scott SE, Walter FM, Webster A (2013). The model of pathways to treatment: conceptualization and integration with existing theory. Br J Health Psychol.

[20] Walter F, Webster A, Scott S, Emery J (2012). The Andersen Model of Total Patient Delay: a systematic review of its application in cancer diagnosis. J Health Serv Res Policy.

[21] Pettigrew LM, Kumpunen S, Mays N (2020). Primary care networks: the impact of covid-19 and the challenges ahead. BMJ.

[22] Gray DM, Joseph JJ, Olayiwola JN (2020). Strategies for digital care of vulnerable patients in a COVID-19 world—keeping in touch. JAMA Health Forum.

[23] Shachar C, Engel J, Elwyn G (2020). Implications for telehealth in a postpandemic future: regulatory and privacy issues. JAMA.

[24] Williams R, Jenkins DA, Ashcroft DM (2020). Diagnosis of physical and mental health conditions in primary care during the COVID-19 pandemic: a retrospective cohort study. Lancet Public Health.

[25] Velasquez D, Mehrotra A (2020). Health Affairs Blog. Ensuring the growth of telehealth during COVID-19 does not exacerbate disparities in care. https://www.healthaffairs.org/do/10.1377/hblog20200505.591306/full/.

[26] Neves AL, Lawrence-Jones A, Naar L (2020). Multidisciplinary teams must work together to co-develop inclusive digital primary care for older people. Br J Gen Pract.

[27] Mehmi A, Winters D, Newman T, Green J (2020). Remote clinics: a panacea during the pandemic or a promoter of health inequality?. Br J Hosp Med.

[28] Loveday C, Sud A, Jones ME (2021). Prioritisation by fit to mitigate the impact of delays in the 2-week wait colorectal cancer referral pathway during the COVID-19 pandemic: a UK modelling study. Gut.

